# Non-systemic metamorphosis in male millipede appendages: long delayed, reversible effect of an early localized positional marker?

**DOI:** 10.1186/1742-9994-5-5

**Published:** 2008-04-08

**Authors:** Leandro Drago, Giuseppe Fusco, Alessandro Minelli

**Affiliations:** 1 Department of Biology, University of Padova, Via Ugo Bassi 58/B, I-35131 Padova, Italy

## Abstract

**Background:**

The development of specialized appendages involved in sperm transfer in the males of julid millipedes is an extreme case of specialized, complex structures differentiating in a very advanced phase of post-embryonic development. Here, a non-systemic metamorphosis affects the external morphology and the internal anatomy of a trunk double segment only.

**Presentation of the hypothesis:**

We hypothesize that during early (possibly embryonic) development a segmental marker is produced that remains unexploited throughout late embryonic and early post-embryonic development, until, activated by a systemic signal, it finally determines the release of a segmentally localized but anatomically major change.

**Testing the hypothesis:**

Key to testing the hypothesis are (1) the identification of both the putative segmental marker involved in the localization of the legs to be eventually metamorphosed into gonopods and the systemic signal activating it, (2) the identification of the cell population from which the gonopods are built, and (3) a longitudinal study of the marker's expression throughout late embryonic and, possibly, post-embryonic development.

**Implications of the hypothesis:**

Proving the validity of this hypothesis would demonstrate the existence of a cryptic developmental module that will be activated only months, or years, after it has been first laid down during early development. This study also opens a window onto the very poorly explored domain of late expression of developmental genes and molecular control of late developmental events.

## Background

**Figure 1:**
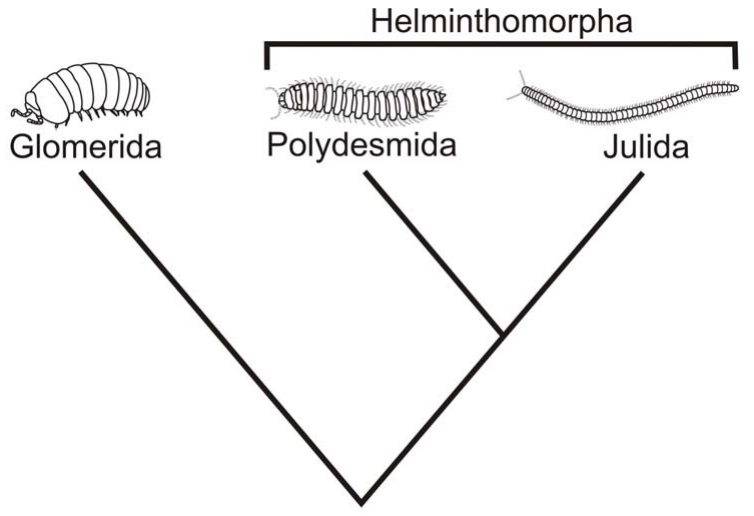
Simplified phylogeny of Diplopoda according to [2], to illustrate the relationship between the taxa cited in the main text.

Translation of positional information into the localized expression of anatomical structures is not limited to the embryonic phase of development, or to a metamorphosis systemically affecting the whole animal body. The development of specialized appendages (gonopods) involved in sperm transfer in the males of julid millipedes (Diplopoda, Julida) [FIG. 1] is an extreme case of specialized, complex and highly species-specific structures differentiating in a very advanced phase of post-embryonic development. This occurs through a metamorphosis that deeply affects external morphology and internal anatomy of the trunk diplosegment bearing the eighth and ninth pair of legs, while leaving unaffected the sections of the trunk that both precede and follow it. We propose to call this kind of dramatic post-embryonic transformations confined to a circumscribed body district *non-systemic metamorphosis*.

Other examples of non-systemic metamorphosis are offered by the development of a new kind of appendages replacing the second and third pair of legs lost by some larval pycnogonids a few instars earlier [1], and by the transformation of the forelegs of cicadas, from the robust digging tools of the nymph into the generalized walking legs of the adult.

In the case of male Helminthomorpha (the large millipede clade that includes the julids), this metamorphosis is limited to one or very few non-terminal serially homologous elements in an otherwise morphologically homogeneous series of trunk segments. In females, no special feature develops at any time at the corresponding location. In some julid species, this localized change is partially reversible, with the occurrence of the peculiar phenomenon called periodomorphosis [2,3]: cyclically, mature males with fully-developed gonopods moult into 'intercalary' males, with these limbs regressed to a condition similar to the small scale-like appendages of the last instar preceding sexual maturity, but resume the condition of reproductive adults with fully-developed gonopods within one or a few more moults.

## Presentation of the hypothesis

**Figure 2:**
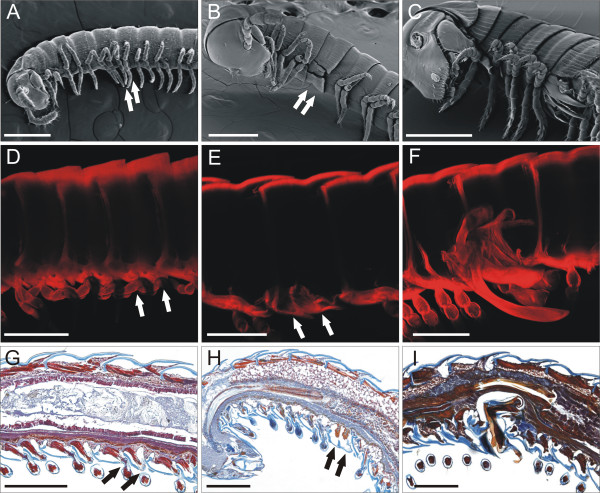
**In the juliform millipede *Nopoiulus kochii *(Gervais, 1847) (Blaniulidae), the eighth and the ninth pair of trunk appendages are typical legs since their first appearance in the III post-embryonic instar and so remain in the IV instar (A, D, G), and only turn into short, scale-like appendages with the moult to the V instar (B, E, H). **With a further moult, the millipede, now in its VI post-embryonic instar, becomes an adult with two pairs of bulky, complex gonopods (C, F, I). (A-C) External morphology. SEM, scale bar 300 μm. (D-F) Esoskeletal and endoskeletal components. Projection at maximum intensity of serial pictures collected with Confocal Laser Scanning Microscope, soft tissues digested with KOH 10%, cuticle stained with Blue Evans 0.005%, scale bar 200 μm. (G-I) Internal anatomy. Para-midsagittal paraffin sections (7 μm), Mallory's triple stain, scale bar 200 μm. Arrow pairs point to the eighth and ninth pair of trunk appendages, as walking legs (A, D, G) or scale-like appendages (B, E, H).

The precise localization of the metamorphic event eventually giving rise to the millipede male gonopods, with the accompanying changes in the overall arrangement of the internal organs in the corresponding segments [FIG. 2], must depend on the strict localization of a positional marker.

We hypothesize that (1) a segmental marker is produced during early (possibly embryonic) development, although a later reinforcement of the signal through novel expression is probable; (2) this marker remains unexploited throughout late embryonic and early post-embryonic development, until it finally determines the transcription of genes or the activation of gene products that release a segmentally localized but anatomically major change.

That this position is marked since early post-embryonic development is suggested, in particular, by the conservation of the position across the Helminthomorpha, in spite of the diversity in post-embryonic segmentation schedules, both as number of segments already formed at hatching and, more conspicuously, as number of segments that are visibly added at each post-embryonic moult, a figure that in some lineages also varies intra-specifically. Thus, although, in principle, a previously unspecified position could subsequently acquire its distinctive identity through signals from sources localized elsewhere in the body, this seems unlikely to occur during millipede post-embryonic development.

The positional mark may be provided by the sustained transcription of a key factor, perhaps assisted by trithorax-like proteins [4], or by the localized expression, determined by an earlier factor, of components of a signal transduction pathway, which make tissues competent to respond to a subsequent non-localized signal, as for instance a hormone.

## Testing the hypothesis

It seems sensible to start the search for a positional marker by a candidate gene approach. The segmental position of gonopods would perhaps suggest that one of the posterior-group *Hox *genes is involved. At present, due to the incomplete expression data for posterior *Hox *genes in helminthomorph millipedes, the only term of comparison would be the pill millipede *Glomeris marginata *[5], where the *Hox *complex respects standard collinearity. This is of limited interest, however, as male pill millipedes do not possess gonopods, although they differentiate, at late post-embryonic instars, the terminal leg-pair as claspers (telopods).

Position and nature of the specified organs might suggest to focus on *Abdominal-B *(*Abd-B*), as this gene is consistently involved in the specification of sexual structures in animals as different as spiders [6], nematodes [7] and mammals [8]. The expression pattern of *Abd-B *has not been studied in the only helminthomorph millipede, the polydesmoid *Oxidus gracilis*, for which data on *Hox *gene expression are available [9]; nevertheless, in this species the anterior border of expression domains of the other posterior-class *Hox *genes is known to occur, embryonically, at positions anterior to those of the gonopods.

In parallel with establishing the molecular nature of the putative segmental marker involved in the localization of the legs to be eventually metamorphosed into gonopods, and identifying the signal that activates it (or releases its suppression), key to testing our hypothesis are (1) the identification of the cell population from which the gonopods are built, followed by (2) a longitudinal study of the marker's expression throughout late embryonic and, possibly, post-embryonic development.

No study is available on the origin, localization, proliferation and differentiation of the cell population eventually giving rise to the gonopods in any millipede. The possible occurrence of small clusters of set-aside cells, comparable to insect histoblasts, should be considered. While their existence would reinforce our suggestion for an early positional specification, this would also open alternative scenarios as to the mechanism involved.

To date, millipedes have been proved to be difficult subjects for the study of development. The few classic studies of their embryonic development [e.g., [10,11]] have been extended by gene expression work on pill millipedes [6,12] and by the already mentioned, very limited account on *Oxidus *[9]. Post-embryonic stages of helminthomorph millipedes are difficult to handle experimentally, because of the heavily calcified armour that covers their body, often completely, and also because of the disturbance to histochemical procedures caused by the diversity of repugnatory substances they produce.

## Implications of the hypothesis

Proving the validity of this hypothesis would demonstrate the existence of a cryptic developmental module that will be activated only months, or years, after it has been first laid down during early development and also the existence of positional markers whose information can even be used repeatedly (periodomorphosis), following morphogenetic reversals of a magnitude and significance only comparable to the loss and regeneration of major body parts.

In evolutionary perspective, this change cannot be equated to the change from the thoracic legs of a caterpillar to the thoracic legs of a butterfly, because in the latter case it is the adult appendage which is phylogenetically conservative, while the larval leg is evolutionarily more derived. In the helminthomorph millipede, what is conserved is the appendage found in the early developmental stages, followed by metamorphosis into a derived one.

The evolution of gonopods is thus recapitulative, in so far as the development of the derived condition of the appendages is preceded by the full development of the primitive condition of the same structures. It is not easy to explain this costly developmental pathway in terms of adaptation. Generally, the legs that are to become gonopods are functionally lost (transformed into scale-like appendages) within one or two moults. On the other hand, an uninterrupted series of typical leg pairs, including those that will later metamorphose into gonopods, is observed since the first post-embryonic instar in a species (*Pachyjulus flavipes*) that hatches with more than twenty leg pairs, rather than the more usual three pairs only [2]. Thus, gonopod evolution in millipedes possibly represent a case of developmental constraints, whose precise nature needs be investigated at molecular level.

This study can also open a window onto the very poorly explored domain of late expression of developmental genes and molecular control of late developmental events.

## Competing interests

The author(s) declare that they have no competing interests.

## Authors' contributions

LD carried out morphological investigations on non-systemic metamorphosis in *Nopoiulus kochii*. All authors participated in the design of the study, in the formulation of the hypothesis, and read and approved the final manuscript.

## References

[B1] Dogiel V (1913). Embryologische Studien an Pantopoden. Ztschr wiss Zool.

[B2] Enghoff H, Dohle W, Blower JG (1993). Anamorphosis in millipedes (Diplopoda). The present state of knowledge and phylogenetic considerations. Zool J Linn Soc.

[B3] Sahli F, Minelli A (1990). On post-adult moults in Julida (Myriapoda, Diplopoda). Why periodomorphosis and intercalaries occur in males?. Proceedings of the 7th International Congress of Myriapodology.

[B4] Simon JA, Tamkun JW (2002). Programming off and on states in chromatin: mechanisms of Polycomb an trithorax group complexes. Curr Opin Genet Dev.

[B5] Janssen R, Damen WG (2006). The ten Hox genes of the millipede *Glomeris marginata*. Dev Gen Evol.

[B6] Damen WGM, Tautz D (1999). *Abdominal-B *expression in a spider suggests a general role for *Abdominal-B *in specifying the genital structures. J Exp Zool.

[B7] Kagoshima H, Cassata G, Bürglin TR (1999). A *Caenorhabditis elegans *homeobox gene expressed in the male tail, a link between pattern formation and sexual dimorphism?. Dev Gen Evol.

[B8] Kondo T, Zakany J, Innis JW, Duboule D (1997). Of fingers, toes and penises. Science.

[B9] Abzhanov A, Popadic A, Kaufman TC (1999). Chelicerate Hox genes and the homology of arthropod segments. Evol Dev.

[B10] Pflugfelder O (1932). Über den Mechanismus der Segmentbildung bei der Embryonalentwicklung und Anamorphose von *Platyrrhacus amauros *Attems. Ztschr wiss Zool.

[B11] Dohle W (1964). Die Entwicklung von *Glomeris marginata *(Villers) im Vergleich zur Entwicklung anderer Diplopoden. Zool Jahrb Anat.

[B12] Janssen R, Prpic NM, Damen WGM (2004). Gene expression suggests decoupled dorsal and ventral segmentation in the millipede *Glomeris marginata *(Myriapoda: Diplopoda). Dev Biol.

